# Supplementary material for: Soil fertility mapping of a cultivated area in Resunga Municipality, Gulmi, Nepal
Source: PLoS One. 2024 Jan 31;19(1):e0292181. doi: 10.1371/journal.pone.0292181 (PMC10830040; doi:10.1371/journal.pone.0292181)
Supplement: S1 File — (a) Citrus farm in one of the soil sampling areas; (b) Farmers with a citrus plant in the study area; (c) Handheld GPS used in the field; (d) Soil sample collection and profile study; (e) Preparation of laboratory equipment; (f) Preparation of soil samples; (g) Laboratory analysis of soil samples. (PDF) [file pone.0292181.s001.pdf]

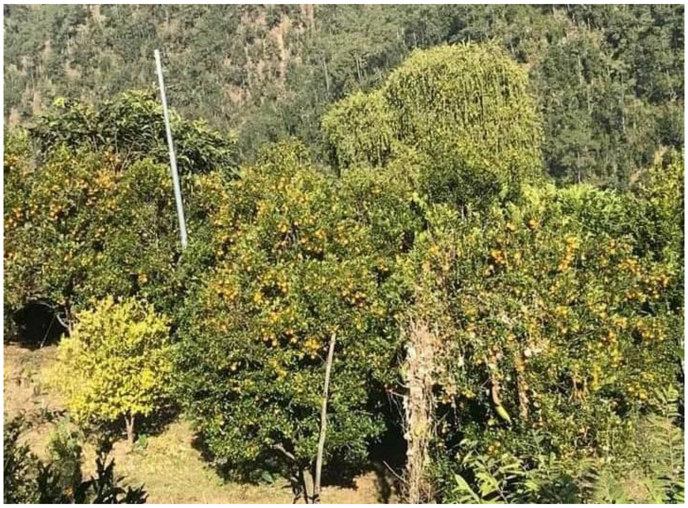

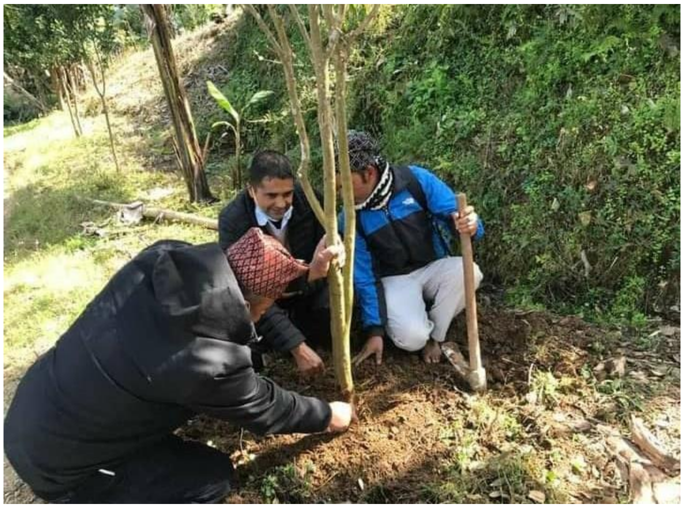

(**a**) (**b**)

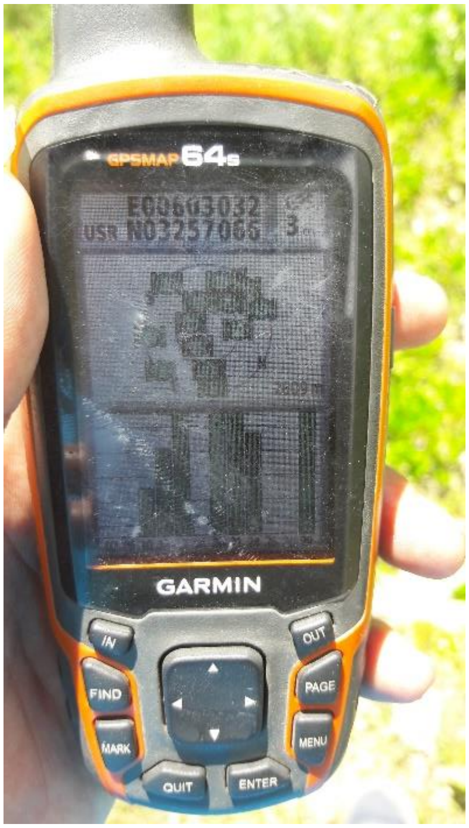

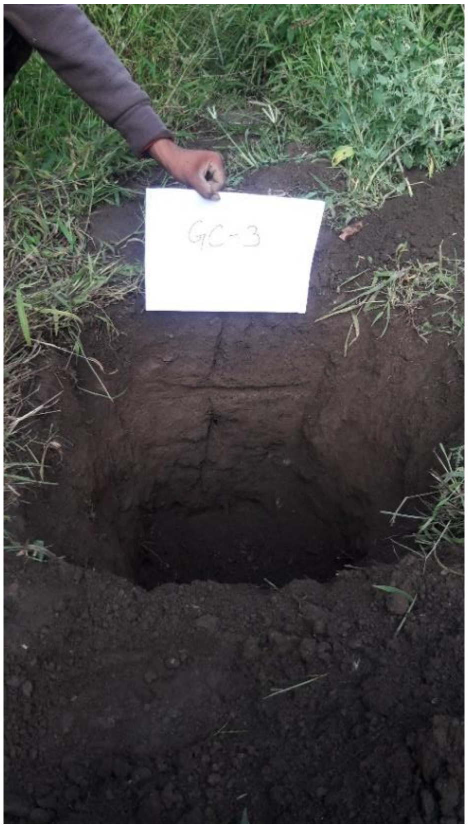

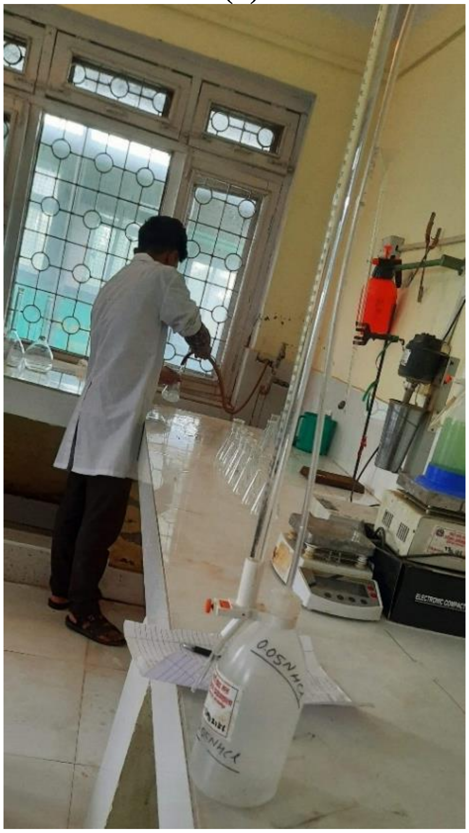

(**c**) (**d**) (**e**)

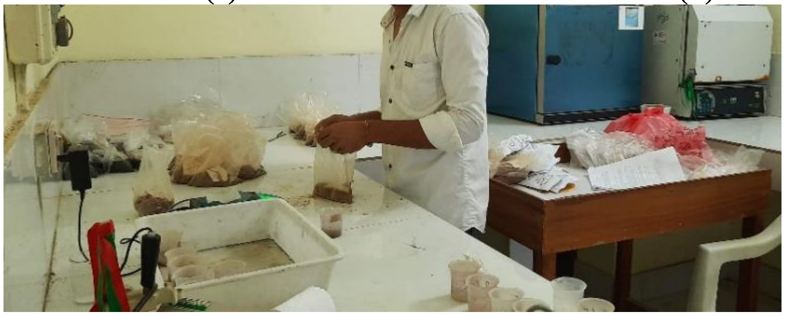

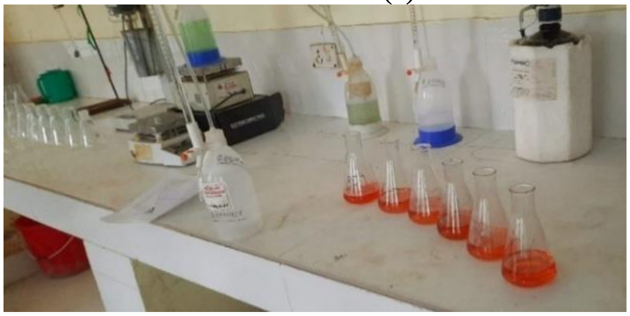

(**f**) (**g**)

S1 Fig. Soil fertility mapping activities in Resunga Municipality, Gulmi, Nepal. (PDF) (**a**) Citrus farm in one of the soil sampling areas; (**b**) Farmers with a citrus plant in the study area; (**c**) Handheld GPS used in the field; (**d**) Soil sample collection and profile study; (**e**) Preparation of laboratory equipment; (**f**) Preparation of soil samples; (**g**) Laboratory analysis of soil samples.
