# Supplementary material for: Soil fertility mapping of a cultivated area in Resunga Municipality, Gulmi, Nepal
Source: PLoS One. 2024 Jan 31;19(1):e0292181. doi: 10.1371/journal.pone.0292181 (PMC10830040; doi:10.1371/journal.pone.0292181)
Supplement: Attachment — Submitted filename: PONE-D-23-01627.pdf [file pone.0292181.s003.pdf]

PLOS ONE

Soil Fertility Mapping of a Cultivated Area in Resunga Municipality, Gulmi, Nepal

--Manuscript Draft--

**Manuscript Number:**	PONE-D-23-01627
**Article Type:**	Research Article
**Full Title:**	Soil Fertility Mapping of a Cultivated Area in Resunga Municipality, Gulmi, Nepal
**Short Title:**	Soil Fertility Mapping of a Cultivated Area in Resunga Municipality, Gulmi, Nepal
**Corresponding Author:**	TRI DEV ACHARYA<br>University of California Davis<br>Davis, CA UNITED STATES
**Keywords:**	soil fertility; soil map; GIS; soil quality; soil nutrients; kriging
**Abstract:**	Soil fertility maps are crucial for sustainable soil and land use management system for predicting soil health status. This study aimed to develop the soil fertility map of agricultural areas in Resunga Municipality, Gulmi district of Nepal using the geographical information system (GIS) technique. A total of 57 composite geo-referenced soil samples from the depth (0-20 cm) were taken from the agricultural land of an area of 52 km2. Soil samples were analyzed for their texture, pH, organic matter, total nitrogen, available phosphorous, available potassium, available boron, and available zinc status. These parameters were modelled to develop the soil quality index. Using the kriging tool, those obtained parameters were interpolated and digital maps were produced along with soil quality and nutrient indices. The result showed that the study area lies within the fair (0.4 to 0.6) and good (0.6 to 0.8) range of the soil quality index representing 96% and 3% respectively. Soil organic matter and nitrogen showed moderate variability exhibiting a low status in 95% and 86% of the total study area. Phosphorous and potassium showed medium status in 88% and 75% of the study area, respectively. Zinc was low and boron status was medium in most of the area. Soil fertility maps provide researchers, farmers, students, and land use planners with easier decision-making tools for sustainable crop production systems and land use management systems.
**Order of Authors:**	Prabin Ghimire, Masters
	Santosh Shrestha, Masters
	Ashok Acharya, Masters
	Aayushma Wagle, Masters
	TRI DEV ACHARYA, PhD
**Opposed Reviewers:**	
**Additional Information:**	
**Question**	**Response**
**Financial Disclosure**<br><br>Enter a financial disclosure statement that describes the sources of funding for the work included in this submission. Review the submission guidelines for detailed requirements. View published research articles from *PLOS ONE* for specific examples.<br><br>This statement is required for submission and **will appear in the published article** if	The author(s) received no specific funding for this work.

Powered by Editorial Manager® and ProduXion Manager® from Aries Systems Corporation

the submission is accepted. Please make sure it is accurate.<br><br>**Unfunded studies**<br>Enter: *The author(s) received no specific funding for this work.*<br><br>**Funded studies**<br>Enter a statement with the following details:<br>• Initials of the authors who received each award<br>• Grant numbers awarded to each author<br>• The full name of each funder<br>• URL of each funder website<br>• Did the sponsors or funders play any role in the study design, data collection and analysis, decision to publish, or preparation of the manuscript?<br>• **NO** - Include this sentence at the end of your statement: *The funders had no role in study design, data collection and analysis, decision to publish, or preparation of the manuscript.*<br>• **YES** - Specify the role(s) played.<br><br>* typeset	
**Competing Interests**<br><br>Use the instructions below to enter a competing interest statement for this submission. On behalf of all authors, disclose any competing interests that could be perceived to bias this work—acknowledging all financial support and any other relevant financial or non-financial competing interests.<br><br>This statement is required for submission and **will appear in the published article** if the submission is accepted. Please make sure it is accurate and that any funding sources listed in your Funding Information later in the submission form are also declared in your Financial Disclosure statement.<br><br>View published research articles from *PLOS ONE* for specific examples.	The authors have declared that no competing interests exist.

Powered by Editorial Manager® and ProduXion Manager® from Aries Systems Corporation

**NO authors have competing interests**<br><br>Enter: *The authors have declared that no competing interests exist.*<br><br>**Authors with competing interests**<br><br>Enter competing interest details beginning with this statement:<br><br>*I have read the journal's policy and the authors of this manuscript have the following competing interests: [insert competing interests here]*<br><br>* typeset	
**Ethics Statement**<br><br>Enter an ethics statement for this submission. This statement is required if the study involved:<br><br>• Human participants<br>• Human specimens or tissue<br>• Vertebrate animals or cephalopods<br>• Vertebrate embryos or tissues<br>• Field research<br><br>Write "N/A" if the submission does not require an ethics statement.<br><br>General guidance is provided below. Consult the submission guidelines for detailed instructions. **Make sure that all information entered here is included in the Methods section of the manuscript.**	N/A

**Format for specific study types**<br><br>**Human Subject Research (involving human participants and/or tissue)**<br>• Give the name of the institutional review board or ethics committee that approved the study<br>• Include the approval number and/or a statement indicating approval of this research<br>• Indicate the form of consent obtained (written/oral) or the reason that consent was not obtained (e.g. the data were analyzed anonymously)<br><br>**Animal Research (involving vertebrate animals, embryos or tissues)**<br>• Provide the name of the Institutional Animal Care and Use Committee (IACUC) or other relevant ethics board that reviewed the study protocol, and indicate whether they approved this research or granted a formal waiver of ethical approval<br>• Include an approval number if one was obtained<br>• If the study involved *non-human primates*, add *additional details* about animal welfare and steps taken to ameliorate suffering<br>• If anesthesia, euthanasia, or any kind of animal sacrifice is part of the study, include briefly which substances and/or methods were applied<br><br>**Field Research**<br><br>Include the following details if this study involves the collection of plant, animal, or other materials from a natural setting:<br>• Field permit number<br>• Name of the institution or relevant body that granted permission	
**Data Availability**<br><br>Authors are required to make all data underlying the findings described fully available, without restriction, and from the time of publication. PLOS allows rare exceptions to address legal and ethical concerns. See the PLOS Data Policy and FAQ for detailed information.	No - some restrictions will apply

A Data Availability Statement describing where the data can be found is required at submission. Your answers to this question constitute the Data Availability Statement and **will be published in the article**, if accepted.<br><br>**Important:** Stating 'data available on request from the author' is not sufficient. If your data are only available upon request, select 'No' for the first question and explain your exceptional situation in the text box.<br><br>Do the authors confirm that all data underlying the findings described in their manuscript are fully available without restriction?	
**Describe where the data may be found in full sentences. If you are copying our sample text, replace any instances of XXX with the appropriate details.**<br><br>• If the data are **held or will be held in a public repository**, include URLs, accession numbers or DOIs. If this information will only be available after acceptance, indicate this by ticking the box below. For example: *All XXX files are available from the XXX database (accession number(s) XXX, XXX.).*<br>• If the data are all contained **within the manuscript and/or Supporting Information files**, enter the following: *All relevant data are within the manuscript and its Supporting Information files.*<br>• If neither of these applies but you are able to provide **details of access elsewhere**, with or without limitations, please do so. For example:<br><br>*Data cannot be shared publicly because of [XXX]. Data are available from the XXX Institutional Data Access / Ethics Committee (contact via XXX) for researchers who meet the criteria for access to confidential data.*<br><br>*The data underlying the results presented in the study are available from (include the name of the third party*	With the permission of Resunga Municipality, Nepal soil sampling data can be made available by the first author. Other results and maps generated during this study are included in the article.

and contact information or URL).<br>• This text is appropriate if the data are owned by a third party and authors do not have permission to share the data.<br><br>* typeset	
Additional data availability information:	

Powered by Editorial Manager® and ProduXion Manager® from Aries Systems Corporation

Cover Letter for submission

To
Editors,
Editorial office, PLOS One

Dear Sir/Madam,

We would like to submit an article, entitled **"Soil Fertility Mapping of a Cultivated Area in Resunga Municipality, Gulmi, Nepal"** as a candidate for publication on ***Plos One***.

Related Classifications of study: Geo-informatics, Soil, Mapping/Modeling, Agriculture.

Abstract of the paper: Soil fertility and quality maps are crucial for sustainable soil and land use management system for predicting soil health status. The objective of this study was to develop the soil fertility and quality map of agricultural areas in Resunga Municipality, Gulmi district of Nepal using the geographical information system (GIS) technique. A total of 57 geo-referenced soil samples from the depth (0-20 cm) were taken from the agricultural land of an area of 52 km2. Soil samples were analyzed for texture, pH, organic matter, nitrogen, phosphorous, potassium, boron, and zinc status. These parameters were modelled to develop the soil quality index. Using the kriging tool, those obtained parameters were interpolated and digital maps were produced along with soil quality and nutrient indices. The result showed that the study area lies within the fair (0.4 to 0.6) and good (0.6 to 0.8) range of the soil quality index representing 96% and 3% respectively. Soil organic matter and nitrogen showed moderate variability exhibiting a low status in 95% and 86% of the total study area. Phosphorous and potassium showed medium status in 88% and 75% of the study area. Zinc was low and boron status was medium in most of the area. Soil fertility and quality map provide researchers, farmers, students, and land use planners with easier decision-making tools for sustainable crop production systems and soil and land use management systems.

Keywords: soil map; soil health; soil nutrients; soil fertility; kriging; GIS.

Information on previous and concurrent submissions: This paper is an extended work of **IECL2022** (1st International Electronic Conference on Land —Towards Land System Science) which is just held on May 17-19, 2022 online. We acknowledge this as a result of Soil survey work from the Department of Soil Science, Institute of Agriculture and Animal Science, Tribhuvan University, Nepal, and Agriculture Knowledge Center, Gulmi, Nepal. It is our original unpublished work, and it has not been submitted to any other journal for review.

Sincerely,

Tri Dev Acharya
Corresponding author on behalf of all authors

Manuscript

Click here to access/download;Manuscript;PLOS_One_SoilFerMap_2023010

Original Manuscript

Soil Fertility Mapping of a Cultivated Area in Resunga Municipality, Gulmi, Nepal

Prabin Ghimire [1], **Santosh Shrestha** [1,2], **Ashok Acharya** [3], **Aayushma Wagle** [4], **Tri Dev Acharya** [5,*]

[1] Department of Soil Science, Institute of Agriculture and Animal Science, Tribhuvan University, Kathmandu, Nepal

[2] Agricultural Technology Center, Lalitpur, Nepal

[3] Department of Soil Science and Agri-engineering, Agriculture and Forestry University, Chitwan, Nepal

[4] Department of Agronomy, Agriculture and Forestry University, Chitwan, Nepal

[5] Institute of Transportation Studies, University of California Davis, Davis, California, USA

[*] Corresponding author

Email: tdacharya@ucdavis.edu (T.D.A.)

Abstract

Soil fertility maps are crucial for sustainable soil and land use management system for predicting soil health status. This study aimed to develop the soil fertility map of agricultural areas in Resunga Municipality, Gulmi district of Nepal using the geographical information system (GIS) technique. A total of 57 composite geo-referenced soil samples from the depth (0-20 cm) were taken from the agricultural land of an area of 52 km^2. Soil samples were analyzed for their texture, pH, organic matter, total nitrogen, available phosphorous, available potassium, available boron, and available zinc ~~status~~. These parameters were modelled to develop ~~the~~ soil quality index. Using the kriging tool, those obtained parameters were interpolated and digital maps were produced along with soil quality and nutrient indices. The result showed that the study area lies within the fair (0.4 to 0.6) and good (0.6 to 0.8) range of the soil quality index representing 96% and 3% respectively. Soil organic matter and nitrogen showed moderate variability exhibiting a low status in 95% and 86% of the total study area. Phosphorous and potassium showed medium status in 88% and 75% of the study area, respectively. Zinc was low and boron status was medium in most of the area. Soil fertility maps provide researchers, farmers, students, and land use planners with easier decision-making tools for sustainable crop production systems and land use management systems.

Introduction

Land and soil have directly or indirectly been included in the global policy framework of the United Nations (UN) Sustainable Development Goals (SDGs). Sustainable soil management is an important aspect of achieving the SDGs, among which ten targets have a direct relation to soil. Soil quality improvement is requisite for attaining the SDGs, specifically the goals of zero hunger (SDG 2), climate action (SDG 13), and life on land (SDG 15) [1]. The unavailability of soil-based indicators assigned to soil-related SDGs is a problem. So, there is a need for soil-based indicators from signalling, design, implementation, and evaluation to achieve the SDGs [2]. Soil fertility and quality refer to the nutrient-supplying ability of soil which is the important aspect that defines the soil and agricultural productivity[3]. Furthermore, land degradation (SDG 15.3) is the consequence of the loss of soil productivity [4]. The set of measurable attributes termed indicators including the nutrient status and environmental condition can be used for monitoring and evaluating soil fertility[5].

The measurable indicators like nutrient status, organic carbon are suggested to achieve the soil-related SDGs target to implement resilient agricultural practices for increasing production and productivity and maintaining the ecosystem and

strengthening the capacity for climate change adaptation which progressively improves the land and soil by 2030, ensuring the sustainable food production system [1].

Soil fertility and quality determination and assessments are essential for the optimization and sustainability of the agroecosystem [6]. It plays important role in land use planning, resource management, and site analysis [7]. Soil data that includes physical and chemical properties are needed to access and monitor which impact the soil fertility and quality with an emphasis on nutrient status and organic carbon to achieve the SDGs[8]. DSM provides detailed work and can be managed and stored within a geographic information system (GIS). The traditional soil survey and mapping is based on the recognition of soil properties and their quantitative relationship within the landscape and environmental variables on conceptual models[9] The implementation of sustainable land use management requires soil mapping[10] and digital soil mapping (DSM) uses an integration of soil data and its environmental covariates. It requires field sampling, laboratory analysis, and remote sensing arranging in various spatial and temporal scales by adopting the appropriate support system[9].

There is a need the provide specific and detailed soil information and use suitable geostatistical methods to study the spatial structures. The use of techniques of spatial interpolation known as kriging to estimate the variable at the unsampled location. Kriging provides an unbiased and linear estimate of variables, and the most common use of kriging is ordinary kriging, it testifies that geostatistical interpolation techniques can be used for the regional level even without ancillary data if there is sufficient data within the study area[11].

Nepal is an agricultural country and soil resources are considered an integral part of its economy. Variability in soil fertility and quality is found across Nepal mainly due to its forming factors such as parent material, climate, topography, and soil organisms[12]. Despite the advancement of digital soil mapping around the world, there are narrative reports and soil maps in the analogue format generated from the majority of soil survey work in Nepal. Soil and Terrain (SOTER) database by Food and Agriculture Organization (FAO) in collaboration with the Nepal survey department has developed a database at the scale of 1: million. This database lacks the details to be used at local levels by farmers. The soil fertility and quality assessment were performed on a random soil sampling method and obtained a qualitative fertility value in farmers' fields [13]. These studies lack spatial variability, and the randomness of variation differs from place to place[14].

This study tries to fill the gap of high-resolution maps with spatial distribution in soil physical and chemical properties of Resunga Municipality. The simple and widely used methodology of ordinary kriging for interpolation of soil nutrient variability can be used to develop the soil nutrient availability map and generate the fertility index. The map produced will

immensely benefit the farmers and local authorities for sustainable soil and land use management and will eventually aid in obtaining the SDGs.

Materials and Methods

Study Area

The study area, Figure 1, was the Resunga municipality of Gulmi district, Nepal. It lies between 637-2338 masl with a total 85 km^2 area and approximately 52 km^2 and 32 km^2 of agricultural and forested area respectively. It experiences an annual average rainfall of 1395 mm and a temperature range of 14-40℃. The study area had citrus cultivation, maize-seasonal vegetables in upland areas, and rice-wheat-maize in lowlands. This region is affected by rapid urbanization, agricultural land is being gradually reduced and transformed into construction sites. The preparation of a soil fertility map shows the present status of agricultural land and can provide the key information for its protection and the reclamation for sustainable land management to ensure sustainable crop production.

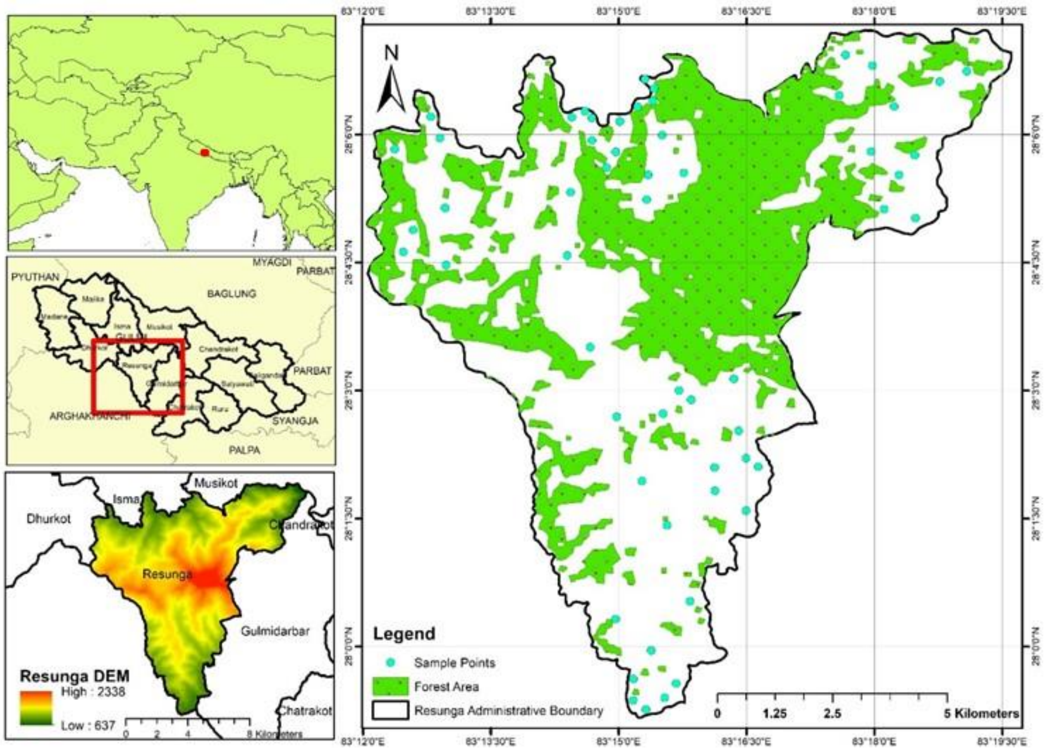

Figure 1. Location map of Resunga municipality of Gulmi district, Nepal as a study area.

Soil Sampling

Sample points were identified from the agricultural area only as this area is a key space for agricultural production rather than a forested area. Sample points were allocated based on slope, aspect, and elevation of the area, and then sampling was done randomly such that a proportionate quantity of sample points gets allocated to each land of Resunga municipality (Figure 2a). Garmin GPS device (Figure 2b) was used to reach the points and samples were taken from 0-20 cm depth with help of a soil auger and a profile study was also performed (Figure 2c). A total of 57 samples were taken from the agricultural area only.

Collected soil samples were prepared and analyzed at the regional soil and fertilizer testing laboratory, Kanchanpur, Mahendranagar, Nepal for routine parameters i.e., soil pH, soil texture, total nitrogen, available phosphorus and potassium, soil organic matter, and micronutrients such as boron and zinc following methods given in Table 1.

Table 1. Soil parameters were tested, and methodology was adopted.

S. N.	Parameter	Unit	Methods
1	Total nitrogen	%	Kjeldahl method [15]
2	Available phosphorus	Ppm	Modified Olsen's Bicarbonate Method [16]
3	Available potassium	~~Ppm~~	Neutral Ammonium Acetate method [17]
4	Soil pH	-	1:2.5 Potentiometric method [17]
5	Soil organic matter	%	Walkley Black method [18]
6	Soil texture	-	Hydrometer method [19]
7	Available boron	~~Ppm~~	Hot water extraction method [20]
8	Available zinc	~~Ppm~~	DTPA extraction method [21]

Note: ppm is parts per million and DTPA is diethylenetriaminepentaacetic acid.

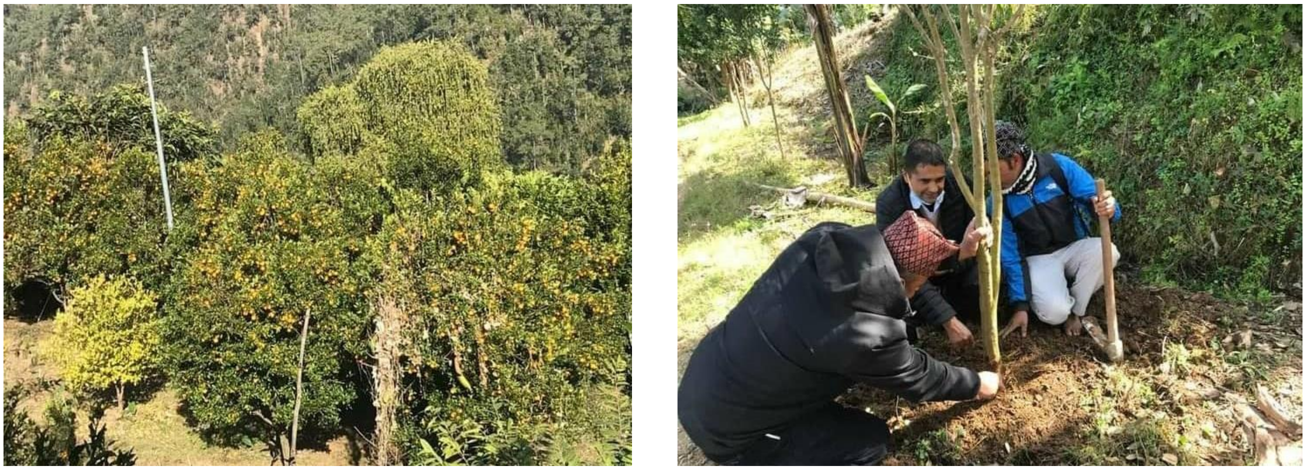

(**a**) (**b**)

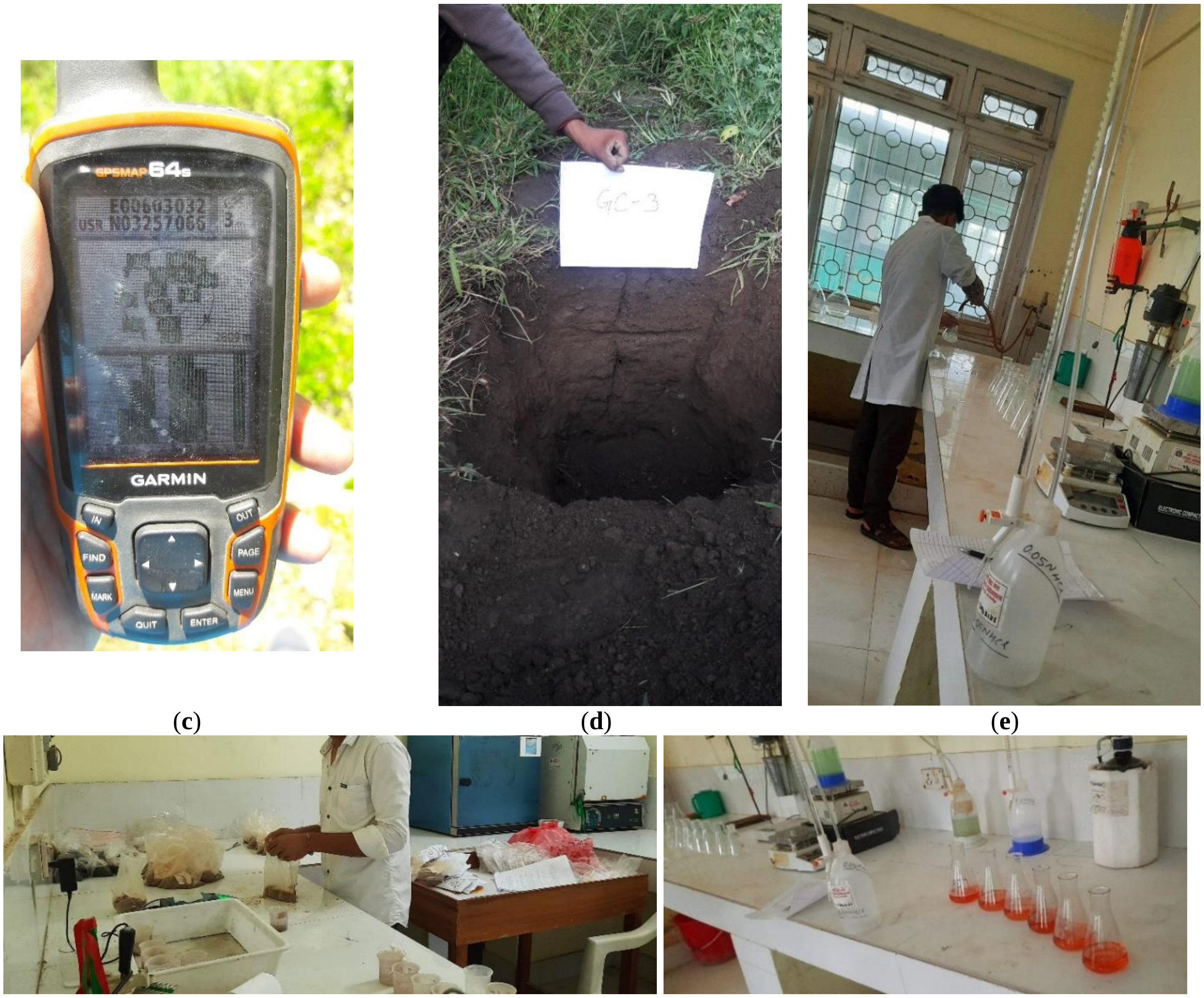

(c) (d) (e)

(f) (g)

Figure 2. Soil fertility mapping activities in Resunga Municipality, Gulmi, Nepal: (**a**) Citrus farm in one of the soil sampling areas; (**b**) Farmers with a citrus plant in the study area; (**c**) Handheld GPS used in the field; (**d**) Soil sample collection and profile study; (**e**) Preparation of laboratory equipment; (**f**) Preparation of soil samples; (**g**) Laboratory analysis of soil samples.

Methodology

Soil chemical properties data of each parameter (pH, OM, N, P_2O_5, K_2O, SQI, B, and Zn) were analyzed for descriptive statistics (mean, median, maximum, minimum, standard deviation standard error of the mean, and coefficient of variation) using R studio 4.0.1. A fertility parameter is considered as weakly variable, moderately variable, or strongly variable when the coefficient of variation (CV%) is < 10,10-100 and >100 respectively[22].

Soil fertility and soil quality map were prepared with Arc GIS 10.8 using interpolation techniques. The ordinary kriging method was deployed in interpolation from the geostatistical wizard for nitrogen, phosphorus, potassium, zinc, boron, pH, and soil quality index. Thiessen polygon was deployed for soil texture map preparation. Figure 3 shows the overall workflow adopted in the study.

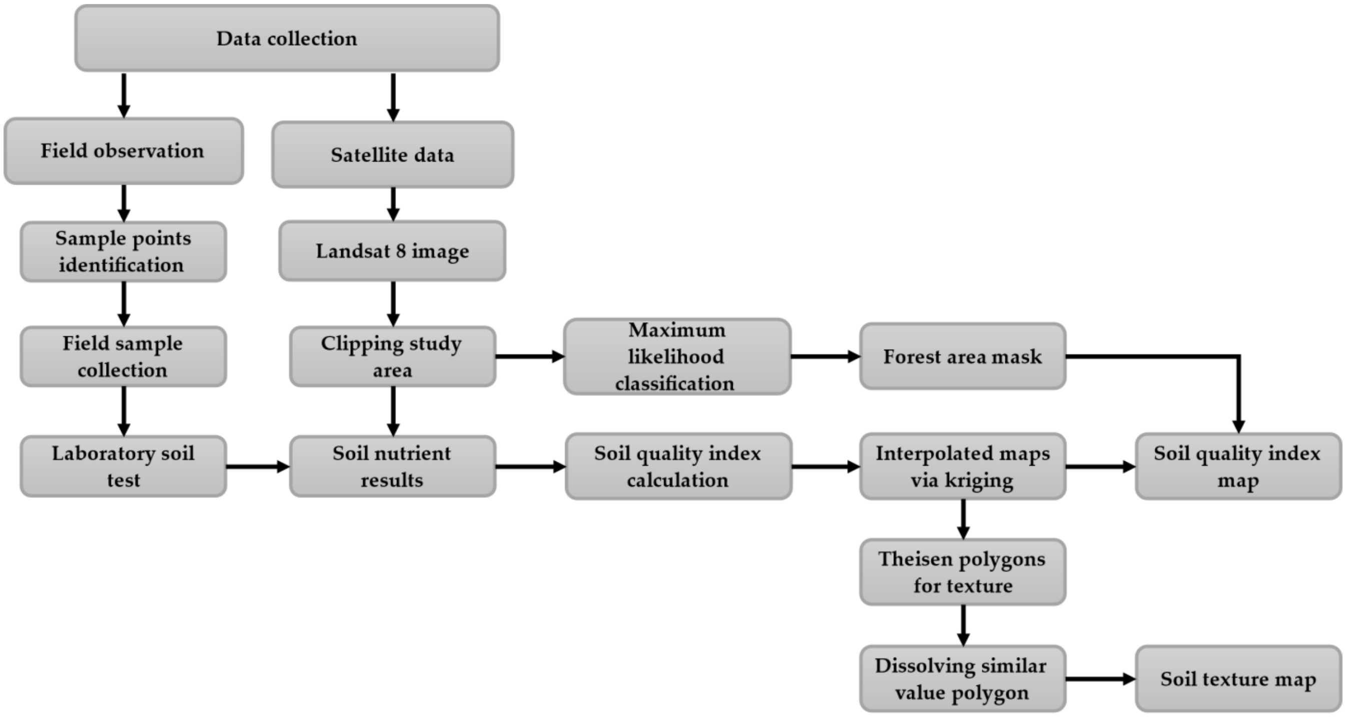

Figure 3. Workflow of the study adopted.

Soil quality index

The soil parameter like available water capacity, infiltration capacity, and aggregate stability are included for the development of the soil quality index [23,24] it is not used in this study due to the unavailability of resources and equipment. So, the soil quality rating (SQR) system was developed by Bajracharya *et al* [25] for the four major soil parameters by adding the product of the weighting factor with assigned parameter ranking values from 0 to 1 (0 is the worst and 1 is best). This is a simple and readily applicable semi-quantitative approach for assessing the overall relative soil quality from an agricultural perspective. Organic matter is considered an important factor as it influences nutrient availability; aggregate stability, erosion susceptibility, etc so it was given the value of 0.4 (c), the major nutrients like N, P, and K are important for crop production so it is valued as 0.3 (d), soil texture as 0.2 (a) and pH is considered the as low degree of importance, but essential soil quality so

valued as 0.1(b). This weighted value for each parameter was done by the Nepal Agriculture Research Council (NARC) and the Department of Agriculture, Nepal [25].

The Soil Quality Index [26] was calculated with the formula given below:

$$SQI = [(a*R_{STC}) + (b*R_{pH}) +(c*R_{oc}) + (d*R_{NPK})] \quad (1)$$

where, a, b, c, and d are the weighted values of each parameter which are 0.2, 0.1, 0.4, and 0.3, respectively.

R_{STC}, R_{pH}, R_{oc}, and R_{NPK} are assigned ranking values of texture, pH, organic matter, and NPK respectively which are given in Table 2. The scoring method developed by the Nepalese Agriculture Research Council (NARC) was used to interpret the soil quality index.

Table 2. Soil quality index ranking values.

**S. N.**	**Parameter**	**Ranking Value**				
		**0.2**	**0.4**	**0.6**	**0.8**	**1.0**
1	Soil fertility (NPK)	Low	Mod. Low	Moderate	Mod. High	High
2	Soil pH	<4, >8.5	4 – 5	5 – 6	6- 6.5, 7.5 - 8.5	6.5-7.5
3	Soil organic matter	≤0.5	0.5 – 1	1 – 2	2 – 4	>4
4	Soil textural class	C, S	CL, SC, SiC	Si, LS	L, SiL, SL	SiCL, SCL
5	Soil Quality Index	V. poor	Poor	Fair	Good	Best

Note: C- Clay, S-Sand, CL-Clay loam, SC- Sandy Clay, SiC- Silty Clay, Si-Silt, LS-Loamy sand, SiL- Silty loam, SL-Sandy loam, LS Loamy Sand, L- loam, SL-Sandy loam, SiCL-Silty clay loam, SCL- Sandy Clay loam.

Soil Nutrient Index (NI)

The soil Nutrient Index (NI) [27,28] was calculated with the given formula:

$$NI = \{(1*A) +(2*B)+(3*C)\}/TNS, \quad (2)$$

where, number of samples in low categories, number of samples in medium categories, number of samples in high categories, TNS= Total number of samples. The value ranges from low (<1.67), medium (1.67-2.33) to high (>2.33).

Global Moran Index

The global moran index was used to calculate the spatial autocorrelation and it is based on feature location and feature values. It evaluates whether the pattern is clustered, dispersed, or random. It utilizes the z-score and p-value to evaluate the significance of the index[29]

Results

Descriptive Statistics of soil properties

The descriptive statistical summary for pH, organic matter, nitrogen, phosphorous, soil quality index, boron, and zinc is presented in Table 3. The coefficient of variation (CV) is used to interpret the variability. The CV ranges from 6.30% in pH to 100.09% in P2O5. There is a different degree of heterogeneity among the soil properties in the study area.

Table 3. Descriptive statistics soil physiochemical status of Resunga Municipality, Gulmi, Nepal.

**Descriptive Statistics**	**Soil Physiochemical Parameters**										
	**N**	**P_2O_5**	**K_2O**	**pH**	**Organic Matter**	**B**	**Zn**	**SQI**	**Sand**	**Silt**	**Clay**
	**%**	**Ppm**	**Ppm**		**%**	**ppm**	**ppm**		**%**	**%**	**%**
Minimum	0.006	0.66	13.61	5.30	0.09	0.04	0.01	0.28	4.8	25.8	20.8
Maximum	0.19	120.72	346.34	7.50	2.48	1.41	0.42	0.76	46.7	50.3	52.6
Mean	0.08	20.96	101.38	6.80	1.23	0.44	0.11	0.52	26.26	36.3	37.45
Median	0.09	15.344	79.56	6.90	1.29	0.41	0.09	0.54	24.8	33.6	38.2
Standard Deviation (SD)	0.03	20.98	73.33	0.43	0.4	0.33	0.07	0.1	11.46	6.19	7.99
Standard Error of Mean (SEM)	0.005	2.78	9.71	0.06	0.06	0.04	0.009	0.01	1.51	0.82	1.06
CV %	40.83	100.09	72.33	6.30	34.13	74.57	67.31	19.97	43.65	17.06	21.34

Table 4. Areas of soil categories based on soil physio-chemical properties.

Parameter	Unit	Range	Class	Area (ha)	% of the Total Area
**Soil texture**			Loam	11.21	13%
			Clay loam	35.99	42%
			Silty clay loam	6.68	8%
			Silty clay	16.88	20%
			Clay	14.34	17%
**N**	%	<0.05	Very low	-	-
		0.05–0.10	Low	81	95%
		0.10–0.20	Medium	3.67	4%
		0.20–0.40	High	-	-
		>0.40	Very high	-	-
**P_2O_5**	ppm	<4	Very low	-	-
		4-13.0	Low	0.07	-
		13-49	Medium	74.91	88.02%
		25-49	High	9.69	11.38%

		>49	Very high	-	-
**K_2O**	ppm	<25	Very low	0.09	0
		25-49	Low	2.39	3%
		49-125	Medium	63.56	75%
		125-223	High	16.61	20%
		>223	Very high	2.01	2%
**pH**		<4.5	Strongly acidic	0.10	-
		4.5-5.5	Moderately acidic	0.90	1%
		5.5-6.5	Slightly acidic	6.00	7%
		6.5-7.5	Neutral	77.67	91%
		>7.5	Strongly alkaline	-	-
**OM**	%	<1	Very low	11.12	13%
		1–2.5	Low	73.56	86%
		2.5–5.0	Medium	-	-
		5.0–10.0	High	-	-
		>10.0	Very high	-	-
**B**	ppm	<0.2	Very low	1.74	2%
		0.2–0.5	Low	36.22	43%
		0.5–1	Medium	46.70	55%
		1–2	High	-	-
		>2.0	Very high	-	-
**Zn**	ppm	<0.25	Very low	80.06	94.07%
		0.25–0.5	Low	-	-
		0.5–1.0	Medium	-	-
		1.0–2.0	High	-	-
		>2.0	Very high	-	-
**Soil Quality**		<0.2	Very poor	-	-
		0.2-0.4	Poor	-	-
		0.4-0.6	Fair	82.01	96%
		0.6-0.8	Good	2.66	3%
		0.8-1	Best	-	-

Spatial variability and map of soil properties

Total nitrogen

The concentration of N varied from 0.006% to 0.19% having a mean value of 0.08$\pm$0.005% (Table 3). N status was low (0.05-0.01%) in 95% of the study area (Table 4) (Figure 3a). The observed variability for total nitrogen content was moderate (40.83%) (Table 3).

Available phosphorous

The concentration of available P_2O_5 ranged from 0.66 to 120.72 ppm with a mean value of 20.96±2.78 ppm% (Table 3). Medium status (11-49 ppm) of available phosphorus in 88.02% of the area followed by high (25-49 ppm) in 11.38% of the study area was observed (Figure 3b) (Table 4). Available P_2O_5 showed strong variability (100.09%) among the studied soil samples.

Available potassium

The concentration of available K_2O ranged from 13.61 to 346.34 ppm with a mean of 101.38±9.71 ppm (Table 3). This indicates a medium (49-125 ppm) to high (125-223 ppm) status of available potassium in most of the area (Table 4) (Figure 3c). The studied soil samples were moderate in variability (72.33%) (Table 3).

Soil pH

The soil pH range varied from 5.30 to 7.50 having a mean of 6.80±0.06 (Table 3). the observed soil pH of the study area is slightly acidic representing 7% to neutral 91% of the total study area (Table 4) (Figure 3d). The soil pH showed weak variability (6.30%) among the studied samples.

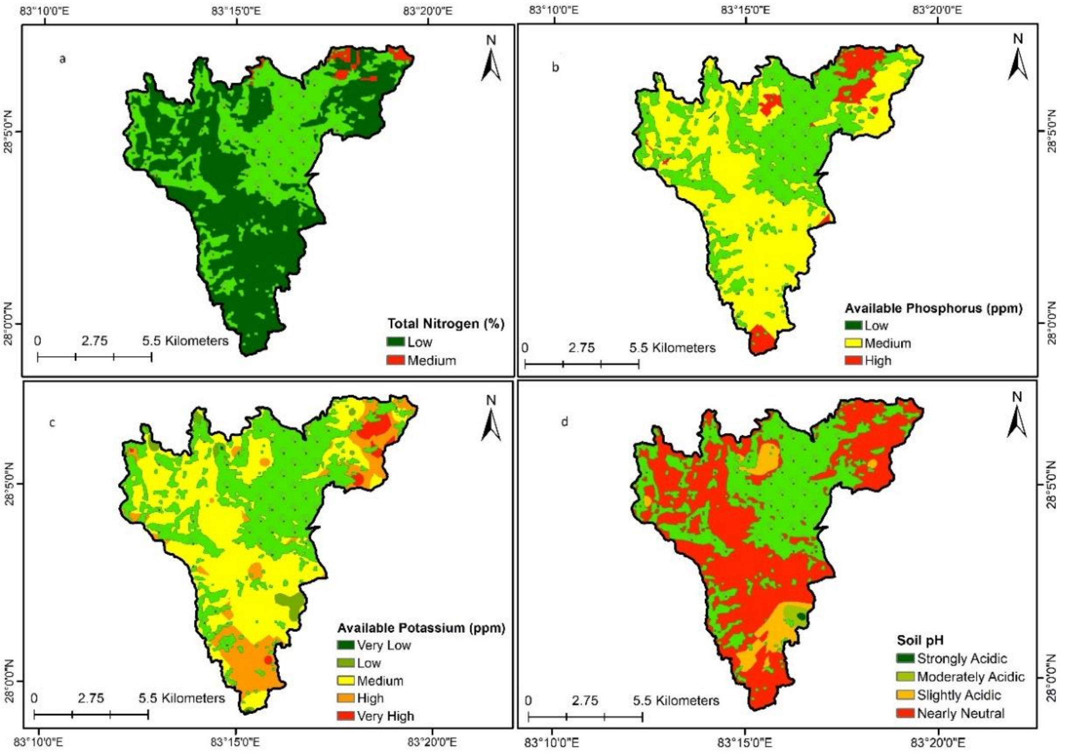

Figure 3. Spatial variability and map of soil properties: (a) Total Nitrogen, (b) Available phosphorous, (c) Available potassium, and (d) soil pH.

Soil organic matter

The concentration of organic matter in all samples have in the range of 0.09 to 2.48% with a mean value of 1.23$\pm$0.06% (Table 3). This indicates very low (<1%) in 13% of the study area to low (1 -2.5%) status organic matter in 86%of the study areas (Figure 4a) (Table 4). The studied samples revealed the organic matter content in the soil has moderate variability of 34.13%.

Soil texture

The sand content in the studied samples ranged from 4 to 46% with a mean of 26.26$\pm$1.51%. Silt content was 25 to 50% with a mean of 36.3$\pm$0.82% while clay content ranged from 20 to 52% with a mean of 37.45$\pm$1.06% (Table 3). The five textural classes; loam, clay loam, silty clay loam, silty clay, and clay were determined, and most of the area contained clay loam (Figure 4b). The moderate variability of sand, silt, and clay were studied at 43.65%, 17.06%, and 21.34% respectively (Table 4).

Available boron

The concentration of boron ranged from 0.04 to 1.41 ppm with a mean of 0.44$\pm$0.04 ppm (Table 3). This exhibits low (0.2-0.5 ppm) to medium (0.5-1ppm) content of available boron in 43% and 55% of the study area respectively (Table 4) (Figure 4c). Moderate variability of 74.75% was seen in the available boron concentration in sampled soil.

Available zinc

The concentration of available zinc t ranged from 0.01 to 0.42 ppm with a mean of 0.11$\pm$0.009 ppm (Table 3). The observation was a very low (<0.25 ppm) status of available zinc throughout the study area (Table 4) (Figure 4d). The variability status was moderate (67.31%) in the sampled soil.

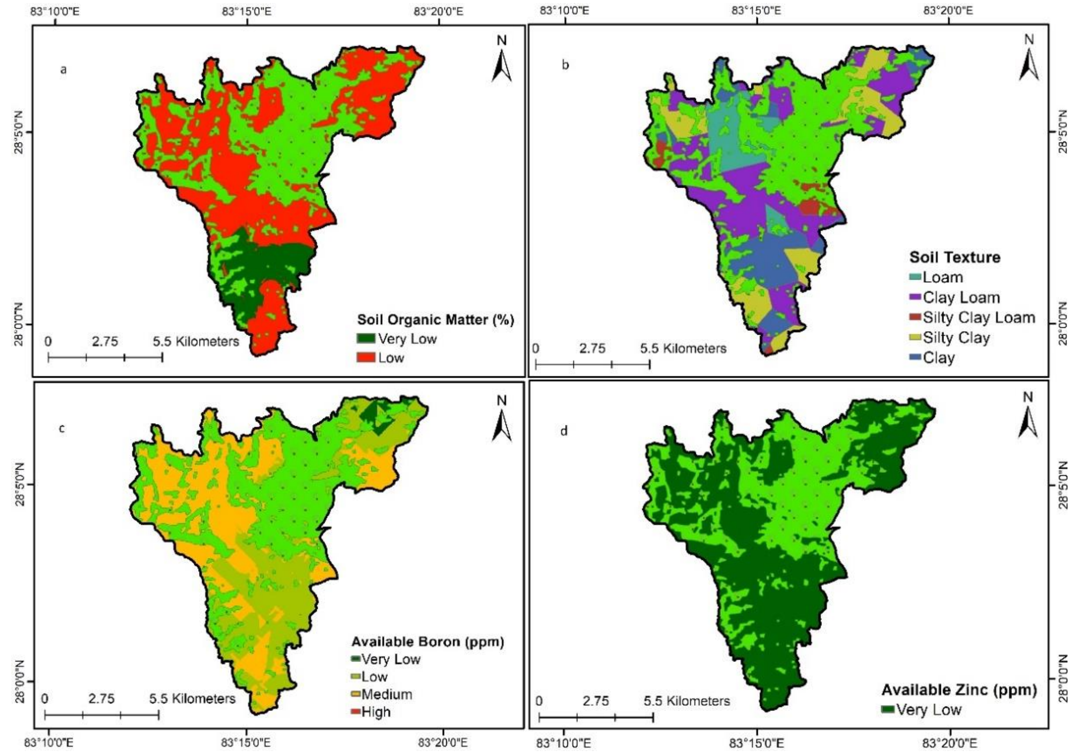

Figure 4. Spatial variability and map of soil properties: (a) Soil organic matter, (b) Soil texture, (c) Available boron, and (d) Available zinc.

Soil quality index

The analysis of the soil quality index (SQI)in all samples exhibited in the range of 0.28 to 0.76 with a mean of 0.52±0.01 (Table 4). This shows fair (0.4-0.6) to good (0.6-0.8) soil quality status in 96% and 3% of the study area respectively (Table 5) (Figure 5). The soil quality index showed moderate variability (19.97%) in the studied soil samples.

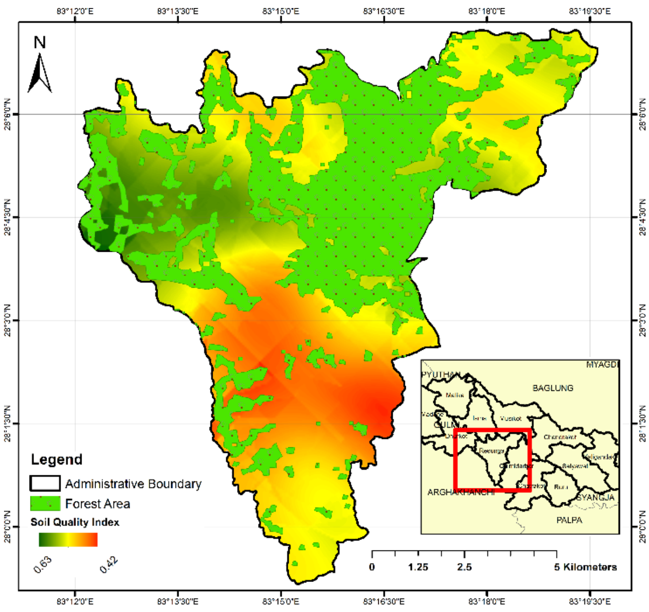

Figure 5. Soil quality index spatial variability map.

Nutrient index

The nutrient index (NI) of N, P, K, and B are 1.368, 1.842, 2.053, and 1.439 respectively. However, the nutrient index for soil organic matter and zinc was least i.e., 1. After calculating the nutrient index and comparing it to the nutrient index rating, the result shows that the soil samples were low in total nitrogen, soil organic matter, boron, and zinc; medium in available phosphorus and available potassium.

Table 5. Nutrient Index data for the nutrient status of Resunga Municipality, Gulmi, Nepal.

S. N.	Parameter	Total Sample nT	Nutrient Index NI	Remarks
1	Total nitrogen	57	1.368	Low
2	Available phosphorus	57	1.842	Medium
3	Available potassium	57	2.053	Medium
4	SOM	57	1	Low
5	Available boron	57	1.439	Low
6	Available zinc	57	1	Low

Note: If NI<1.67, low; NI from 1.67-2.33, medium; NI>2.33, high.

Spatial autocorrelation

Global Moran's I Index is used for the calculation of the spatial pattern of soil chemical properties (Table 6). The distribution of soil chemical properties was random throughout the study area is the hypothesis for the global Moran's I index analysis. With the small *p*-value ($p<0.005$), and the z score of very high or very low ($1.96<z$ and $z<-1.96$), a random form of distribution is not observed in the spatial pattern. the neighbouring values are similar and have a spatial dependency when Moran's I Index value is positive. A negative Moran's I Index value suggests that the neighbouring values are dissimilar and have a negative spatial dependency. Moran's I Index value of 0 suggests a lack of spatial pattern [30,31]. Organic matter and P_2O_5 have a negative Moran's I Index, while all other soil chemical properties have a positive Moran's I Index for their spatial distribution. The spatial distribution of pH, OM, N, P_2O_5, K_2O, Zn, and B did not observe a significantly different from a random distribution at $p<0.005$.

Table 6. Test of significance of pattern analysis of selected parameters.

Parameters	pH	OM	N	P2O5	K2O	Zn	B
Moran's I Index	0.112	-0.011	0.025	-0.042	0.0081	0.037	0.050
Variance	0.006	0.0068	0.0068	0.0069	0.007	0.0062	0.007
Z-Score	1.586	0.007	-0.088	-0.291	0.310	0.703	0.819
P-Value	0.112	0.941	0.929	0.77	0.756	0.481	0.412

Discussion

Soil pH influences the soil's physio-chemical, and biological properties and impacts plant growth and yield [32]. The adoption of different soil management practices could be the reason for a variation in the soil pH. Furthermore, the variability in the soil pH could be due to the nature of parent materials, microtopography, and the types and duration of fertilizer used [33]. Soil acidity is caused by the release of H+ ions during the transformation and cycling of carbon, nitrogen, sulfur, and fertilizer reactions [34]. The normal pH range for optimal mineral element availability and sustained crop production is 6.0 to 7.5 [35]. The majority of cropland in Nepal has less than 2.2% of organic matter status [36]. The low status of organic matter is due to inappropriate adaptation of sustainable soil management practices. The above-ground crop residue is used as animal feed which then affects its incorporation into the soil [37]. Incorporating the crop residue in the soil increases the fertility and organic matter percentages[38]. The increased use of chemical fertilizer application has not balanced with the application of organic matter to date rate of organic matter application was 2.5 to 3 tons ha^{-1} for fertility management in Nepal [39].

Furthermore, the mineralization rate also due to high temperature can cause low organic matter in the soil. The low status of soil organic matter is an indication of soil degradation and soil erosion [40].

The variability in the total N content is due to the variation in soil management practices, application of fertilizer, and Farm Yard Manure (FYM) in the study area. The deficiency in the total soil nitrogen is mainly due to low organic matter content, mineralization, and inadequate application of nitrogen to high nitrogen-demanding plants [33]. Nitrogen content is significantly low in the cultivated area compared to forest and grazing land [41]. Nitrogen is highly susceptible to loss through ammonia volatilization, runoff, and leaching [42]. Soil phosphorous is most affected by soil pH, the ideal value between 6 and 7.5 is best for phosphorous availability[43] while pH below 5.5 and between 7.5 and 8.5 decreases phosphorous availability in soil due to aluminium, iron, or calcium fixation. Phosphorous does not leach out of the root zone, erosion and runoff are the possible causes of P loss. The application of organic matter and placement of phosphorus fertilizer also impact the availability of phosphorus[44]. The availability and variability of phosphorous depend upon mean annual temperature (MAT) and mean annual precipitation (MAP), soil available phosphorous decreases significantly with increasing MAT and MAP[45].

The medium to high status of potassium may be due to the low availability of calcium ions in the soil and the overall pH status of the soil was slightly acidic to neutral (5.3 to 7.5) with a variability percentage of 6.30%. The soil acidity is crucial for the availability of potassium in the soil thus reducing the calcium and magnesium ion in the soil [46]. The application of organic fertilizer and phosphorous has significantly increased the availability of potassium in soil [47]. The zinc and boron status of Nepalese soils is low in concentration and its deficiency is widespread and acute [48]. A similar type of variability in soil properties is also observed in research conducted in Zimbabwe, where variation in soil properties ranges from 11.94% to 121.99% [22].

B is available in the form of uncharged boric acid or borate to plants depending upon the soil conditions like soil moisture, temperature, pH, salinity, organic matter, and rainfall [49]. Boron deficiency is recorded as a problem and accounts for 80-90%of Nepalese soil. Zinc deficiency is widespread, and it affects 20-50 %of agricultural land in Nepal[48]. The B and Zn deficiency in Nepal is due to the mismanagement of the soil rather than a general deficiency[12]. The variable affecting the micronutrient availability is pH, zinc availability rises with the decreasing pH[50,51] and boron becomes less available with increasing pH [52].

Soil quality indices help to determine the soil quality of a particular location or ecosystem and enable the comparison between different management practices and land use[53]. The SQI was assessed so that the management issues are not only

focused on crop productivity but soil degradation and environmental problems [54]. Soil quality may be affected by land use and agricultural management practices due to alterations in the physical, chemical, and biological properties of soil which results in a change in productivity [55].

The result of the study shows that there is spatial variability in soil fertility parameters across the Resunga Municipality. These variations have direct implications for sustainable soil management. This type of intensive soil sampling, analysis, and mapping should be conducted at the ward level at regular intervals to get a clear picture of the soil fertility status of farmland. In the case of highly variable soil properties, there is the uncertainty of field scale estimates so a field-specific recommendation for small landholders is not feasible[56].

Conclusions

Descriptive statistics and soil fertility and soil quality map using interpolation technique were used to determine the spatial variability of soil nutrient status of agricultural land (0-20 cm depth) of Resunga Municipality. Based on sampled data, maps of soil fertility and quality were found to be useful in describing the spatiality of soil attributes in non-sampled sites. The ordinary kriging method in interpolation from the geostatistical wizard is effective for determining the spatial variability of soil nutrients and is recommended for further soil mapping. Soil nutrients and quality maps aid farmers, scientists, and students in decision-making based on existing soil conditions and recommend soil test-based fertilizer recommendations for intensive and sustainable crop cultivation. Soil quality and fertility maps are useful for site-specific nutrient management and soil health monitoring for SDGs and must be developed for all ward and municipality levels for sustainable present and future agricultural approaches.

Author Contributions

Conceptualization: Prabin Ghimire, Ashok Acharya

Data curation: Prabin Ghimire, Santosh Shrestha

Formal analysis: Prabin Ghimire, Santosh Shrestha, Tri Dev Acharya

Investigation: Aayushma Wagle

Methodology: Prabin Ghimire, Santosh Shrestha, Ashok Acharya

Software: Prabin Ghimire

Supervision: Tri Dev Acharya

Validation: Santosh Shrestha

Visualization: Prabin Ghimire, Santosh Shrestha, Tri Dev Acharya

Writing – original draft: Prabin Ghimire, Aayushma Wagle

Writing – review & editing: Tri Dev Acharya

Funding

This research received no external funding. The APC was funded by the University of California Davis Open Access Fund (UCD-OAF).

Data Availability Statement

With the permission of Resunga Municipality, Nepal soil sampling data can be made available by the first author. Other results and maps generated during this study are included in the article.

Acknowledgements

The authors would like to acknowledge the Agriculture Knowledge Center, Gulmi for providing the technical support during the study and laboratory support by Regional Soil Testing Laboratory Sundarpur, Kanchanpur, Mr. Lalit BC for the technical support, Mr. Saurav Marahatta and Ms. Bala Sharma during the preparation of this manuscript, and #Mentor4Nepal Initiative for providing the support for research guidance and publication.

Conflicts of Interest

The authors declare no conflict of interest.

References

1. Tóth, G.; Hermann, T.; da Silva, M.R.; Montanarella, L. Monitoring Soil for Sustainable Development and Land Degradation Neutrality. *Environmental Monitoring and Assessment* **2018**, *190*, 1–4, doi:10.1007/S10661-017-6415-3/TABLES/1.

2. Bouma, J.; Montanarella, L. Facing Policy Challenges with Inter- and Transdisciplinary Soil Research Focused on the Un Sustainable Development Goals. *SOIL* **2016**, *2*, 135–145, doi:10.5194/SOIL-2-135-2016.

3. Karlen, D.L.; Kovar, J.L.; Cambardella, C.A.; Colvin, T.S. Thirty-Year Tillage Effects on Crop Yield and Soil Fertility Indicators. *Soil and Tillage Research* **2013**, *130*, 24–41, doi:10.1016/J.STILL.2013.02.003.

4. 2000 World Day to Combat Desertification | UNCCD Available online: https://www.unccd.int/events/other/2000-world-day-combat-desertification (accessed on 12 March 2022).

5. Panwar, P.; Pal, S.; Reza, S.K.; Sharma, B. Soil Fertility Index, Soil Evaluation Factor, and Microbial Indices under Different Land Uses in Acidic Soil of Humid Subtropical India. *http://dx.doi.org/10.1080/00103624.2011.622820* **2011**, doi:10.1080/00103624.2011.622820.

6. Yang, M.; Mouazen, A.; Zhao, X.; Guo, X. Assessment of a Soil Fertility Index Using Visible and Near-Infrared Spectroscopy in the Rice Paddy Region of Southern China. *European Journal of Soil Science* **2020**, *71*, 615–626, doi:10.1111/EJSS.12907.

7. Lee, E.M.; Griffiths, J.S. The Importance of Pedological Soil Survey in Land Use Planning, Resource Assessment and Site Investigation. *Geological Society, London, Engineering Geology Special Publications* **1987**, *4*, 453–466, doi:10.1144/GSL.ENG.1987.004.01.55.

8. Li, G.; Chen, J.; Sun, Z.; Tan, M. Establishing a Minimum Dataset for Soil Quality Assessment Based on Soil Properties and Land-Use Changes. *Acta Ecologica Sinica* **2007**, *27*, 2715–2724, doi:10.1016/S1872-2032(07)60059-6.

9. Lucà, F.; Buttafuoco, G.; Terranova, O. GIS and Soil. *Comprehensive Geographic Information Systems* **2018**, *3*, 37–50, doi:10.1016/B978-0-12-409548-9.09634-2.

10. Pereira, P.; Brevik, E.C.; Oliva, M.; Estebaranz, F.; Depellegrin, D.; Novara, A.; Cerdà, A.; Menshov, O. Goal Oriented Soil Mapping: Applying Modern Methods Supported by Local Knowledge. *Soil Mapping and*

Process Modeling for Sustainable Land Use Management **2017**, 61–83, doi:10.1016/B978-0-12-805200-6.00003-7.

11. Chilès, J.-P.; Delfiner, P. Geostatistics. **2012**, doi:10.1002/9781118136188().

12. Carson, B. The Land, The Farmer, and The Future; A Soil Fertility Management Strategy for Nepal. **1992**, doi:10.53055/ICIMOD.136.

13. Panday, D. Adapting Climate Change in Agriculture: The Sustainable Way in Nepalese Context. *Hydro Nepal: Journal of Water, Energy and Environment* **2012**, 91–94, doi:10.3126/HN.V11I1.7222.

14. Panday, D.; Ojha, R.B.; Chalise, D.; Das, S.; Twanabasu, B. Spatial Variability of Soil Properties under Different Land Use in the Dang District of Nepal. *Cogent Food & Agriculture* **2019**, *5*, 1600460, doi:10.1080/23311932.2019.1600460.

15. Bremner, J.M.; Mulvaney, C.S. Nitrogen Total. Methods of Soil Analysis. In *American Society of Agronomy*; Page, A.L., Ed.; Madison, WI, USA, 1982; Vol. 9, pp. 595–624.

16. Olsen, S.R.; Cole, C.V.; Watanabe, F.S.; Dean, L.A. *Estimation of Available Phosphorus in Soils by Extraction with Sodium Bicarbonate*; 1954;

17. Jackson, M.L. *Soil Chemical Analysis*; Prentice Hall of India Pvt. Ltd.: New Delhi, India, 1967;

18. Walkley, A.J.; Black, I.A. Estimation of Soil Organic Carbon by the Chromic Acid Titration Method. *Soil Science* **1934**, *37*, 29–38.

19. Bouyoucos, G.J. Hydrometer Method Improved for Making Particle Size Analyses of Soils 1. *Agronomy Journal* **1962**, *54*, 464–465, doi:10.2134/agronj1962.00021962005400050028x.

20. Berger, K.C.; Truog, E. Boron Determination in Soils and Plants Using the Quinalizarin Reaction. *Industrial and Engineering Chemistry - Analytical Edition* **1939**, *11*, 540–545, doi:10.1021/AC50138A007/ASSET/AC50138A007.FP.PNG_V03.

21. Lindsay, W.L.; Norvell, W.A. Development of a DTPA Soil Test for Zinc, Iron, Manganese, and Copper. *Soil Science Society of America Journal* **1978**, *42*, 421–428, doi:10.2136/SSSAJ1978.03615995004200030009X.

22. Soropa, G.; Mbisva, O.M.; Nyamangara, J.; Nyakatawa, E.Z.; Nyapwere, N.; Lark, R.M. Spatial Variability and Mapping of Soil Fertility Status in a High-Potential Smallholder Farming Area under Sub-Humid Conditions in Zimbabwe. *SN Applied Sciences* **2021**, *3*, 396, doi:10.1007/s42452-021-04367-0.

23. Karlen, D.L. Soil Quality as an Indicator of Sustainable Tillage Practices. *Soil Tillage Res* **2004**, *78*, 129–130, doi:10.1016/J.STILL.2004.02.001.

24. Karlen, D.L.; Kovar, J.L.; Cambardella, C.A.; Colvin, T.S. Thirty-Year Tillage Effects on Crop Yield and Soil Fertility Indicators. *Soil Tillage Res* **2013**, *130*, 24–41, doi:10.1016/J.STILL.2013.02.003.

25. Bajracharya, R.M.; Sitaula, B.K.; Sharma, S.; Jeng, A. Soil Quality in the Nepalese Context - An Analytical Review. *International Journal of Ecology and Environmental Sciences* **2007**, *33*, 143–158.

26. Bajracharya, R.M.; Sitaula, B.K.; Sharma, S.; Jeng, A. Soil Quality in the Nepalese Context - An Analytical Review. *International Journal of Ecology and Environmental Sciences* **2007**, *33*, 143–158.

27. Ramamoorthy, B.; Bajaj, J. Available Nitrogen, Phosphorus and Potassium Status of Indian Soils. *Fertilizer News* **1969**, *14*, 25–36.

28. Ravikumar, P.; Somashekar, R.K. Evaluation of Nutrient Index Using Organic Carbon, Available P and Available K Concentrations as a Measure of Soil Fertility in Varahi River Basin, India. *Proceedings of the International Academy of Ecology and Environmental Sciences* **2013**, *3*, 330–343.

29. How Spatial Autocorrelation (Global Moran's I) Works—ArcGIS Pro | Documentation Available online: https://pro.arcgis.com/en/pro-app/latest/tool-reference/spatial-statistics/h-how-spatial-autocorrelation-moran-s-i-spatial-st.htm (accessed on 12 September 2022).

30. Al-Ahmadi, K.; Al-Zahrani, A. Spatial Autocorrelation of Cancer Incidence in Saudi Arabia. *International Journal of Environmental Research and Public Health 2013, Vol. 10, Pages 7207-7228* **2013**, *10*, 7207–7228, doi:10.3390/IJERPH10127207.

31. Saxena, R.; Nagpal, B.N.; Das, M.K.; Srivastava, A.; Gupta, S.K.; Kumar, A.; Jeyaseelan, A.T.; Baraik, V.K. A Spatial Statistical Approach to Analyze Malaria Situation at Micro Level for Priority Control in Ranchi District, Jharkhand. *The Indian journal of medical research* **2012**, *136*, 776–782.

32. Neina, D. The Role of Soil PH in Plant Nutrition and Soil Remediation. *Applied and Environmental Soil Science* **2019**, *2019*, doi:10.1155/2019/5794869.

33. Vasu, D.; Singh, S.K.; Sahu, N.; Tiwary, P.; Chandran, P.; Duraisami, V.P.; Ramamurthy, V.; Lalitha, M.; Kalaiselvi, B. Title: Assessment of Spatial Variability of Soil Properties Using Geospatial Techniques for Farm 1 Level Nutrient Management 2 Authors Name and Affiliations. **2017**.

34. Bolan, N.S.; Curtin, D.; Adriano, D.C. ACIDITY. *Encyclopedia of Soils in the Environment* **2005**, *4*, 11–17, doi:10.1016/B0-12-348530-4/00173-9.

35. Sanchez, P.A.; Palm, C.A.; Buol, S.W. Fertility Capability Soil Classification: A Tool to Help Assess Soil Quality in the Tropics. *Geoderma* **2003**, *114*, 157–185, doi:10.1016/S0016-7061(03)00040-5.

36. Campolo, J.; Güereña, D.; Maharjan, S.; Lobell, D.B. Evaluation of Soil-Dependent Crop Yield Outcomes in Nepal Using Ground and Satellite-Based Approaches. *Field Crops Research* **2021**, *260*, 107987, doi:10.1016/j.fcr.2020.107987.

37. Paudel, G.S.; Thapa, G.B. Changing Farmers' Land Management Practices in the Hills of Nepal. *Environmental Management* **2001**, *28*, 789–803, doi:10.1007/S002670010262.

38. Pandit, K.; Technology, M.B.-N.J. of S. and; 2006, undefined An Assessment of Soil Fertility Management Issues in Pokhare Khola Watershed, Dhading. *researchgate.net*, doi:10.3126/njst.v7i0.578.

39. Khadka, D. Soil Fertility Assessment and Mapping of Chungbang Farm, Pakhribas, Dhankuta, Nepal. *Advances in Plants & Agriculture Research* **2018**, *8*, 219–227, doi:10.15406/apar.2018.08.00317.

40. Zhao, Y.-N.; He, X.-H.; Huang, X.-C.; Zhang, Y.-Q.; Shi, X.-J. Increasing Soil Organic Matter Enhances Inherent Soil Productivity While Offsetting Fertilization Effect under a Rice Cropping System. *Sustainability* **2016**, *8*, 879, doi:10.3390/su8090879.

41. Yimer, F.; Ledin, S.; Abdelkadir, A. Soil Organic Carbon and Total Nitrogen Stocks as Affected by Topographic Aspect and Vegetation in the Bale Mountains, Ethiopia. *Geoderma* **2006**, *135*, 335–344, doi:10.1016/J.GEODERMA.2006.01.005.

42. Ma, Z.; Yue, Y.; Feng, M.; Li, Y.; Ma, X.; Zhao, X.; Wang, S. Mitigation of Ammonia Volatilization and Nitrate Leaching via Loss Control Urea Triggered H-Bond Forces. *Scientific Reports* **2019**, *9*, 15140, doi:10.1038/s41598-019-51566-2.

43. White, P.J.; Brown, P.H. Plant Nutrition for Sustainable Development and Global Health. *Annals of botany* **2010**, *105*, 1073–1080, doi:10.1093/aob/mcq085.

44. Ch'ng, H.Y.; Ahmed, O.H.; Majid, N.M.A. Improving Phosphorus Availability in an Acid Soil Using Organic Amendments Produced from Agroindustrial Wastes. *The Scientific World Journal* **2014**, *2014*, 1–6, doi:10.1155/2014/506356.

45. Hou, E.; Chen, C.; Luo, Y.; Zhou, G.; Kuang, Y.; Zhang, Y.; Heenan, M.; Lu, X.; Wen, D. Effects of Climate on Soil Phosphorus Cycle and Availability in Natural Terrestrial Ecosystems. *Global Change Biology* **2018**, *24*, 3344–3356, doi:10.1111/GCB.14093.

46. Goswami, G.; Deka, P.; Das, P.; Bora, S.S.; Samanta, R.; Boro, R.C.; Barooah, M. Diversity and Functional Properties of Acid-Tolerant Bacteria Isolated from Tea Plantation Soil of Assam. *3 Biotech* **2017**, *7*, 229, doi:10.1007/s13205-017-0864-9.

47. Bader, B.R.; Taban, S.K.; Fahmi, A.H.; Abood, M.A.; Hamdi, G.J. Potassium Availability in Soil Amended with Organic Matter and Phosphorous Fertiliser under Water Stress during Maize (Zea Mays L) Growth. *Journal of the Saudi Society of Agricultural Sciences* **2021**, *20*, 390–394, doi:https://doi.org/10.1016/j.jssas.2021.04.006.

48. Andersen, P. A Review of Micronutrient Problems in the Cultivated Soil of Nepal. *Mountain Research and Development* **2007**, *27*, 331–335, doi:10.1659/mrd.0915.

49. Shorrocks, V. The Occurrence and Correction of Boron Deficiency. *Plant and Soil* **1997**, *193*, 121–148, doi:10.1023/A:1004216126069.

50. Alloway, B.J. Soil Factors Associated with Zinc Deficiency in Crops and Humans. *Environmental Geochemistry and Health* **2009**, *31*, 537–548, doi:10.1007/s10653-009-9255-4.

51. Sillanpää, M.; Vlek, P.L.G. Micronutrients and the Agroecology of Tropical and Mediterranean Regions. In *Micronutrients in Tropical Food Crop Production*; Vlek, P.L.G., Ed.; Springer Netherlands: Dordrecht, 1985; pp. 151–167 ISBN 978-94-009-5055-9.

52. Gupta, U.C, Jame.Y.W, Campbell, C.A, Leyshon, A.J, W.Nicholaichuk. Boron Toxicity and Deficiency:A Review. *Canadian Journal of Soil Science* **1985**, *65*, 381–409.

53. Gelaw, A.M.; Singh, B.R.; Lal, R. Organic Carbon and Nitrogen Associated with Soil Aggregates and Particle Sizes Under Different Land Uses in Tigray, Northern Ethiopia. *Land Degradation & Development* **2015**, *26*, 690–700, doi:10.1002/LDR.2261.

54. Mukherjee, A.; Lal, R. Comparison of Soil Quality Index Using Three Methods. *PLoS ONE* **2014**, *9*, e105981, doi:10.1371/journal.pone.0105981.

55. Islam, K.R.; Weil, R.R. Land Use Effects on Soil Quality in a Tropical Forest Ecosystem of Bangladesh. *Agriculture, Ecosystems & Environment* **2000**, *79*, 9–16, doi:10.1016/S0167-8809(99)00145-0.

56. Schut, A.G.T.; Giller, K.E. Soil-Based, Field-Specific Fertilizer Recommendations Are a Pipe-Dream. *Geoderma* **2020**, *380*, 114680, doi:10.1016/J.GEODERMA.2020.114680.
