# Supplementary material for: Soil fertility mapping of a cultivated area in Resunga Municipality, Gulmi, Nepal
Source: PLoS One. 2024 Jan 31;19(1):e0292181. doi: 10.1371/journal.pone.0292181 (PMC10830040; doi:10.1371/journal.pone.0292181)
Supplement: Attachment — Submitted filename: PONE-D-23-01627_rev1_Reviewer2.docx [file pone.0292181.s004.docx]

**General Comment:** The article investigates Soil Fertility Mapping of a Cultivated Area in Resunga Municipality, Gulmi, Nepal. It is an interesting approach and provide useful information for the readers. However, some improvements are necessary and more details is needed to add so as considered accepted in the PLOS ONE Journal.

**Response:** Dear Dr. AbdelRahman,

Thank you very much for the valuable time to review our manuscript. We very much appreciate the thoughtful suggestions and comments made by the reviewer which we believe have improved the quality of our manuscript.

Being from a least-developed country like Nepal, we faced challenges in finding sufficient literature and data to conduct even simpler research, as one would expect from a developed or information-rich country. Nepal lacks high-resolution soil data despite being an agrarian country. Thus, in this work, we have put forth our utmost effort to develop methodologies that can be base cases and replicated all over the country. As a result, we expect this will encourage other municipalities to produce their high-resolution soil map. Moreover, due to the difficult geography and distance from academic institutions, there is a significant research gap in this nation. We kindly request our reviewers to acknowledge these challenges and help us improve the foundation of research in this field.

Given the importance of this publication, we anticipate that it will become a seminal article in Nepalese universities and serve as a catalyst for further research on soil science within the country.

**Point 1:** Title: The title of the article is accurate and directly relates to the purpose of the research.

**Response 1:** Thank you very much for finding the title to the purpose.

**Point 2:** The abstract should contain a clear statement of recommendations in such studies. I suggest to rewrite the abstract.

**Response 2:** Thank you for the comment. We have followed <https://mitcommlab.mit.edu/cee/commkit/abstract/> as a reference and revised and revised our abstract as follows:

“Soil fertility maps are crucial for sustainable soil and land use management system for predicting soil health status. However, many regions of Nepal lack updated or reliable soil fertility maps. This study aimed to develop the soil fertility map of agricultural areas in Resunga Municipality, Gulmi district of Nepal using the geographical information system (GIS) technique. A total of 57 composite geo-referenced soil samples from the depth (0-20 cm) were taken from the agricultural land of an area of 52 km^2^. Soil samples were analyzed for their texture, pH, organic matter, total nitrogen, available phosphorous, available potassium, available boron, and available zinc. These parameters were modelled to develop a soil quality index (SQI). Using the kriging tool, obtained parameters were interpolated and digital maps were produced along with soil quality and nutrient indices. The result showed that the study area lies within the fair (0.4 to 0.6) and good (0.6 to 0.8) range of SQI representing 96% and 3% respectively. Soil organic matter and nitrogen showed moderate variability exhibiting a low status in 95% and 86% of the total study area. Phosphorous and potassium showed medium status in 88% and 75% of the study area, respectively. Zinc was low and boron status was medium in most of the area. To maintain soil fertility is by improving the rate of exogenous application of fertilizers and manures. The application of micronutrients like boron and zinc is highly recommended in the study area along with organic manures. The soil fertility map can be used as a baseline for soil and land use management in Resunga Municipality. We recommend further studies to validate the map and assess the factors affecting soil fertility in this region. Soil fertility maps provide researchers, farmers, students, and land use planners with easier decision-making tools for sustainable crop production systems and land use management systems.”

**Point 3:** Introduction: Finally, specify the main purpose of the work and highlight the main hypotheses. In its present form, there is a double repetition of the same thread.

In the last paragraph of the introduction clearly state the novelty of the current study, according to the author knowledge and the missing/gap in the literature.

**Response 3:** Thanks for the constructive comment, we have edited the introduction section, especially the last two paragraphs as follows to show the importance of the work and clearly state the objectives:

“Nepal is an agricultural country and soil resources are considered an integral part of its economy. Variability in soil fertility and quality is found across Nepal mainly due to its forming factors such as parent material, climate, topography, and soil organisms [12]. Despite the advancement of digital soil mapping around the world, there are narrative reports and soil maps in the analogue format generated from the majority of soil survey work in Nepal. Soil and Terrain (SOTER) database by Food and Agriculture Organization (FAO) in collaboration with the Nepal survey department has developed a database at the scale of 1: million. This database lacks the details to be used at local levels by farmers. The gap of high-resolution maps with spatial distribution in soil physical and chemical properties can be filled by field sampling and providing descriptive statistics and spatial variability of soil fertility parameters, soil quality and soil nutrient index in the form of maps that will have actual value to the farmers' fields [13]. Nepal lacks these types of studies with detailed spatial variability, as the hilly regions have high variability from place to place [14].

The objective of the study is to perform soil sampling and provide descriptive statistics and spatial variability of soil fertility parameters, soil quality and soil nutrient index in the form of maps of a cultivated area in Resunga Municipality, Gulmi, Nepal. The study uses a simple and widely used methodology of ordinary kriging for interpolation of soil nutrient variability that can be used to develop soil maps. It is the first attempt at high-resolution mapping in the region, The maps produced will immensely benefit the farmers and local authorities for sustainable soil and land use management and will eventually aid in obtaining the SDGs in the region.”

**Point 4:** In the study area chapter add some details about the climate and particularly the annual precipitation and temperature pattern.

**Response 4:** Thanks for the comment, we have added the following text for the climate information:

“It experiences an annual average rainfall of 1395 mm and a temperature range of 14-40℃.”

**Point 5:** Materials and Methods: Refine the class boundaries.

**Response 5:** We apologize that we are not clear on this and thought it might be defining the class boundaries. The class boundaries are taken as per the standard class boundaries in the literature. Most similar works do similar class boundaries but could not find any original reference which defines the class boundaries. If the reviewer has any idea on that then, we welcome that in our next round of review.

**Point 6:** figures 3, and 5 is not readable, legend much enlarge.

**Response 6:** Thanks for the suggestion. We have increased the font size and bolded fonts for clear visualization. We have exported the image with a greater resolution to enhance the quality of all figures. Also, we will provide the editorial with high resolution images for publication.

**Point 7:** Discussion: The discussion is very poor in the context of research in this area by other authors and the proportion in the structure of the work. There are two possible solutions in this case: 1) broadening with the use of the relations already indicated in the Results chapter, 2) Merging the chapters Results and Discussion with the approval of plos one editor.

**Response 7:** Thanks and we very much appreciate the reviewer's suggestion for asking us to separate the discussion and conclusion.

We followed the journal’s guidelines (<https://journals.plos.org/plosone/s/submission-guidelines>) to find out that Results, Discussion, and Conclusions can be merged and separate sections are not mandatory.

“**Results, Discussion, Conclusions**

These sections may all be separate, or may be combined to create a mixed Results/Discussion section (commonly labeled “Results and Discussion”) or a mixed Discussion/Conclusions section (commonly labeled “Discussion”). These sections may be further divided into subsections, each with a concise subheading, as appropriate. These sections have no word limit, but the language should be clear and concise.

Together, these sections should describe the results of the experiments, the interpretation of these results, and the conclusions that can be drawn.

Authors should explain how the results relate to the hypothesis presented as the basis of the study and provide a succinct explanation of the implications of the findings, particularly in relation to previous related studies and potential future directions for research.”

We found another journal with: “Discussions include “Authors should discuss the results and how they can be interpreted in perspective of previous studies and the working hypotheses. The findings and their implications should be discussed in the broadest context possible, and the limitations of the work highlighted. Future research directions may also be mentioned. This section may be combined with Results.”

While further researching, we found that Broad Research Communication Lab’s CommKit suggests a journal article to have a Discussion in the following way without a conclusion. And that matches the journal’s guidelines as well.

Source: <https://mitcommlab.mit.edu/broad/commkit/journal-article-discussion/>

Thus, we used the discussion and conclusion section as follows:

a. Discussion: The first and second paragraph concludes the results and the following paragraphs analyze the results with the ground reality. We have pointed out the thing that can be discussed for the study area and Nepal as a whole for soil nutrient and fertility mapping. The last paragraph includes limitations and a way forward for future research.
b. Conclusion: No conclusion, merged as the first paragraph in the Discussion.

We also looked into a few previous published papers in other open-access SCIE journals without conclusions and took them as references:

- <https://doi.org/10.3390/ijgi10100635> and
- <https://doi.org/10.3390/app10228083>

**Point 8:** Conclusions: The conclusions are constructive. Please just specify the summary of the results as in the abstract (see the manuscripts).

- AbdelRahman M. A. E., Rehab H. H., Yossif T. M. H. (2021) Soil fertility assessment for optimal agricultural use using remote sensing and GIS technologies. Applied Geomatics. <https://doi.org/10.1007/s12518-021-00376-1>
- AbdelRahman, M.A.E., Natarajan, A., C.A. Srinivasamurty & Hegde R. Estimating soil fertility status in physically degraded land using GIS and remote sensing techniques in Chamarajanagar district, Karnataka, India. Egypt. J. Remote Sensing Space Sci. (2016), <http://dx.doi.org/10.1016/j.ejrs.2015.12.002>
- AbdelRahman, M.A.E.; Metwaly, M.M.; Afifi, A.A.; D’Antonio, P.; Scopa, A. Assessment of Soil Fertility Status under Soil Degradation Rate Using Geomatics in West Nile Delta. Land 2022, 11, 1256. <https://doi.org/10.3390/land11081256>.

**Response 8:** We thank the reviewer for pointing out improvements with the references. As we wrote above, we have the first paragraph as a conclusion last as limitations for the study with a way forward.

**Point 9:** Technical Notes: I suggest to create a scheme/figure that better explain the interconnections between the several sections/subsections and between the methodologies proposed and the several results.

**Response 9:** Thank you for the suggestion, we think that the following Fig 2 represents the schematic interconnection of the data used and the flow of work.

**Figure 2.** Workflow of the methodology adopted.

**General Comment:** Recommends that the POLS ONE Editorial Board publish the article taking these minor changes into account.

*Once again, thank you very much for the comments. We have tried our best to modify the manuscript from our understanding and hope this makes the article clearer to the readers. In case of any missing points and improvements, we are very happy to accommodate them in the next round of revisions.*

*Thank you!*
